# Reduced Motor Cortex Deactivation in Individuals Who Suffer from Writer's Cramp

**DOI:** 10.1371/journal.pone.0097561

**Published:** 2014-05-15

**Authors:** Yi-Jhan Tseng, Rou-Shayn Chen, Wan-Yu Hsu, Fu-Jung Hsiao, Yung-Yang Lin

**Affiliations:** 1 Institute of Physiology, National Yang-Ming University, Taipei, Taiwan; 2 Institute of Brain Science, National Yang-Ming University, Taipei, Taiwan; 3 Institute of Clinical Medicine, National Yang-Ming University, Taipei, Taiwan; 4 Laboratory of Neurophysiology, Taipei Veterans General Hospital, Taipei, Taiwan; 5 Integrated Brain Research Laboratory, Taipei Veterans General Hospital, Taipei, Taiwan; 6 Department of Neurology, Taipei Veterans General Hospital, Taipei, Taiwan; 7 Department of Neurology, Chang Gung Memorial Hospital and Chang Gung University College of Medicine, Taipei, Taiwan; 8 Department of Education and Research, Taipei City hospital, Taipei, Taiwan; University of Toronto, Canada

## Abstract

This study investigated the neuromagnetic activities of self-paced finger lifting task and electrical median nerve stimulation in ten writer's cramp patients and fourteen control subjects. The event-related de/synchronizations (ERD/ERS) of beta-band activity levels were evaluated and the somatosensory cortical activity levels were analyzed using equivalent-current dipole modeling. No significant difference between the patients and control subjects was found in the electrical stimulation-induced beta ERS and electrical evoked somatosensory cortical responses. Movement-related beta ERD did not differ between controls and patients. Notably, the amplitude of the beta ERS after termination of finger movement was significantly lower in the patients than in the control subjects. The reduced movement-related beta ERS might reflect an impairment of motor cortex deactivation. In conclusion, a motor dependent dysregulation of the sensorimotor network seems to be involved in the functional impairment of patients with writer's cramp.

## Introduction

Writers' cramp (WC) is a task-specific focal dystonia [1]. Although the exact pathophysiological mechanisms remain unknown, an involvement of the motor cortical areas has been proposed [2], [3]. Excessive movement is involved, which includes abnormal muscle activity, co-contraction of antagonist muscles and over flow of activity into muscles not intended for the task [1]. Abnormal contingent negative variation has been found among patients with WC, suggesting a defect in motor programming [2]. Although disturbance in motor performance is the most apparent manifestation among patients with focal dystonia, sensory trick phenomena have also been observed [4]. Previous studies have demonstrated an alteration in finger representation across the primary somatosensory cortex, together with functional abnormalities in touch localization and temporal discrimination [5], [6]. Abnormalities in sensorimotor integration have also been reported among focal dystonia patients [7]. However, the specific pathophysiological involvement of sensory and motor cortical processing has not been well clarified as yet.

Oscillatory activity in the beta frequency range (13–30 Hz) within the primary sensorimotor cortex of the human brain is observed widely. The power of beta oscillation increases and exceeds the resting level following movement termination [8], [9] or in response to somatosensory stimulation [10], [11]. This phenomenon is called beta event-related synchronization (ERS) and has been suggested to reflect an inhibition of the motor cortex [12] or a sensory reafference [13]. Several sensorimotor-related diseases have been found to involve an impairment of post movement beta rebound [14]–[16]. In 2000, Toro and his colleagues have reported a reduced beta desynchronization in focal hand dystnoia [3]. The author interpreted it as a malfunction of motor cortex. However, the author did not address the post-movement changes of beta activities. Based on the results described above, WC can be characterized as sensorimotor dysfunction. We expect that patients with WC will have aberrant beta oscillatory activity.

The objective of the present study was to investigate the sensorimotor nature of WC by assessing changes of beta oscillatory activities related to voluntary movement and sensory processing. In order to delineate whether the aberrant beta activity is attributed to motor efferent output or by sensory afferent input, movement-related beta ERS during a finger lifting task and somatosensory evoked fields (SEFs) and sensory induced beta ERS were measured.

## Methods

### Subjects

Ten patients with WC (5 men and 5 women; mean age 36.7±14.2 years) and fourteen healthy controls (8 men and 6 women; mean age 31.5±10.8 years) were recruited. All of the subjects were right-handed. Patients with idiopathic WC were clinically evaluated and diagnosed by an experienced neurologist. Table 1 shows the clinical profiles of the patients. Patient 2 had been treated with a Botox injection three months prior to this study. All subjects had been free from any medication for at least two weeks prior to the study. The study protocol was approved by the institutional review board of Taipei Veterans General Hospital and has been performed in accordance with the ethical standards laid down in the 1964 Declaration of Helsinki. Each subject gave written informed consent prior to the study.

**Table 1:** Clinical profiles of the ten writer's cramp patients.

Number	Sex	Age (years)	Handedness	Disease duration (years)	Affected side	Dystonic pattern
1	M	19	Right	1	Right	Extension of 1^st^, flexion of 4^th^ and 5^th^ fingers
2	F	34	Right	10	Right	Forearm extension
3	F	30	Right	14	Right	Wrist flexion and tight grip of pen
4	M	55	Right	23	Right	Wrist flexion and tight grip of pen
5	F	45	Right	9	Right	Abduction of thumb
6	F	40	Right	5	Right	Grip pen tightly
7	M	22	Right	1	Right	Flexion of 4^th^ and 5^th^ fingers
8	M	22	Right	2	Right	Extension of 1^st^ finger
9	F	44	Right	3	Right	Flexion of thumb and 2^nd^ finger
10	M	59	Right	2	Right	Grip pen tightly

Patient 2 received botox injection 3 months before MEG measurement.

### Acquisition of magnetoencephalographic (MEG) data

The MEG recordings were conducted in a magnetically shielded room with a whole-scalp 306-channel neuromagnetometer (Vectorview, Elekta Neuromag, Helsinki, Finland) that consisted of 102 identical triple sensor elements. Each subject was seated comfortably. The recording passband and signal digitization rates were 0.1–160 Hz and 500 Hz, respectively. Responses coincident with prominent vertical electro-oculogram signals (>150 µV) were rejected.

The neuromagnetic responses of finger movement and electrical median nerve stimulation appear mainly around the sensorimotor cortex. N20m and P35m are robust components under median nerve stimulation. We quantified the amount of these SEFs components to estimate the function of sensory processing. These short-latency components of SEF are estimated in the primary somatosensory cortex contralateral to the stimulated site.

Both movement-related and sensory induced beta ERS are predominant over contralateral sensorimotor cortex. Beta ERS over the ipsilateral sensorimotor cortex is varying across participants. Some participants did not have clear ipsilateral beta ERS. Therefore, only the neuromagnetic responses over the contralateral sensorimotor cortex were analyzed in this study.

### Electrical stimulation of the median nerve

Constant 0.2-ms electric pulses were delivered onto the right median nerve at the wrist with an interstimulus interval of 3 s. The intensity of the stimulation was 20% above the motor threshold and it thereby elicited a visible twitch of the thumb. During the MEG recordings, each subject was instructed to remain relaxed and to keep their eyes open. One hundred artifact-free epochs were collected for each subject.

### Analysis of the SEF data

The SEFs were filtered within 0.1∼100 Hz and analyzed with an epoch of 500 ms including a pre-stimulus baseline of 100 ms. SEF deflections were visually assessed to select the time windows and cortical areas of interest for further analysis. We applied an equivalent current dipole (ECD) model to analyze the sources and strengths of the SEFs recorded by the 204 planar gradiometers. Only ECDs for SEFs with a goodness-of-fit value greater than 70% were chosen for subsequent analysis. This approach has been previously described in detail in a number of studies [17]–[20]. N20m was defined as the deflection peaking at about 20 ms after stimulus onset. The P35m response was defined as the subsequent opposite deflection, which typically peaked at around ∼35 ms [19].

### Electromyographic (EMG) recordings and self-paced finger lifting task

A pair of gold disc electrodes was placed at the belly of the right extensor digitorum communis. Each subject was instructed to lift the right index finger approximately once every 8 seconds. In total, 50 artifact-free epochs of index finger lifting were collected for each individual. All patients lifted their index finger without any symptoms.

During a self-paced finger lifting task, the surface EMG signals from the extensor digitorum communis were recorded and amplified 1000 fold with passband 0.1–160 Hz and a signal digitization rate at 500 Hz. To access the timing of EMG onset and offset, offline EMG signals were high-pass filtered at 100 Hz to avoid movement artifacts and subsequently were rectified. The rectified EMG signals were then normalized using a z-transformation. The threshold level was defined as one standard deviation of the normalized EMG data. The EMG signals were analyzed within 100–160 Hz range which was sufficient to detect the EMG onset and offset. Also, the EMG signals were visually examined in order to assess performance of this task and determine the onset and offset of the finger movement.

### Event-related (de)synchronization (ERD/ERS)

Frequency analysis was performed using Matlab 7.0.4 (MathWorks, Natick, MA) and the Fieldtrip toolbox, which was developed at the F. C. Donders Centre for Cognitive Neuroimaging [21]. ERD/ERS calculations resulted in time-frequency maps for the range from 1 to 30 Hz of the percentage band power changes, which were relative to the band power in the reference period, and were calculated separately for each subject. The frequency width of each bin is 1 Hz. Movement-related beta ERD was analyzed within a time window of 4.5 seconds between 3.5 seconds before and 1 second after EMG onset. Movement-related beta ERS was analyzed within a time window of 5.5 seconds between 3.5 seconds before and 2 seconds after EMG offset. The baseline period was from −3.5 to −2.5 seconds. The electrical-induced beta ERS was analyzed within a time window of 2 seconds between 0.5 seconds before and 1.5 seconds after the onset of electrical stimulation. The baseline period was set as 0.5–0.1 seconds before the electrical trigger. Visual inspection of the time-frequency maps was performed for each subject to select the individual reactive 3 Hz beta frequency bands (13–30 Hz). The individual reactive 3 Hz beta-band power was averaged from the 50 artifact-free finger lifting trials carried out by each subject. The beta ERS value within the maximal reactive channel contralateral to the finger lifting hand or electrical median nerve stimulation was selected for statistical analysis.

### Statistical analysis

The data were presented as means ± standard errors of the means (SEM). The Kolmogorov Smirnov test was used to test the normality of the data distribution. The data were non-normally distributed. Therefore, the Mann-Whitney U test, a nonparametric test, was used to compare 2 independent groups. Bonferroni correction was applied when appropriate. Statistical significance threshold was set as *p*<0.05.

## Results

All patients and control subjects completed the task without difficulties. None of the patients experienced writer's cramp symptoms during the finger lifting session. There were no differences in the averaged rectified EMG burst durations between the two groups.

Since botulinum toxin could affect the cortical excitability and plasticity of the central nerve system, the data from patient 2 might confound the results. However, the patient executed the finger lifting task well. Therefore, the data obtained from patient 2 was included in the statistical analysis.

### The source waveforms and location of the SEF

**Figure 1:**
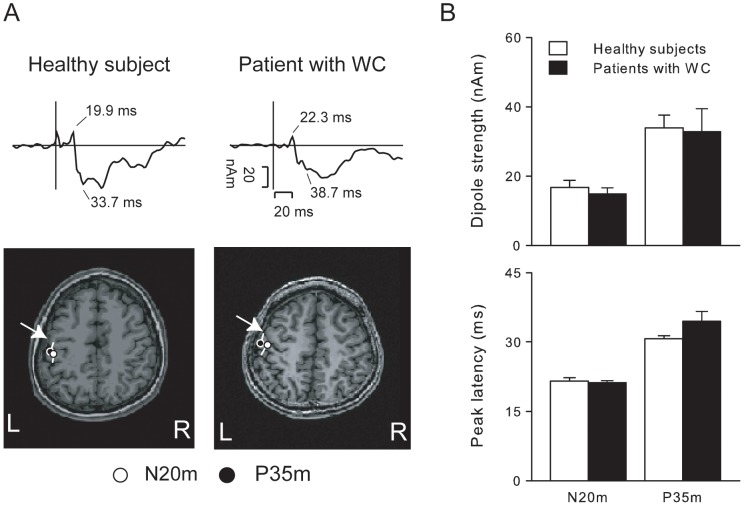
Somatosensory evoked neuromagnetic activities in response to right median nerve stimulation. **A**. Typical waveforms and cortical generators of the left hemispheric SEFs in one healthy subject and one patient. Upper panel: the responses were evoked after the stimulus onset (vertical lines). Lower panel: the ECD localization of N20m (black dots) and P35m (white dots) components superimposed on the subject's own MR images. N20m and P35m have opposite orientations. **B**. The mean dipole strength and peak latency of the N20m and P35m responses in fourteen control subjects and ten patients with WC. Error bars indicate the standard error of the mean (SEM). The arrow indicates the location of the central sulcus. R, right; L, left; WC, writer's cramp.

The source waveforms of the early SEFs on right median nerve stimulation from one healthy subject and from one patient are presented in Fig. 1A. The earliest deflection peaking is around ∼20 ms and was followed by a signal of opposite polarity at around ∼35 ms. The N20m was oriented primarily in the posterior–anterior direction, whereas the opposite orientation was found for P35m. The sources of both the N20m and the P35m response were located in the posterior wall of the central sulcus, which is in line with earlier studies [19], [22], [23]. The strengths of N20m and P35m responses were not different between the controls and patients. Furthermore, the peak latencies of the SEF component did not differ between the two groups (see Fig. 1B). No subject complained of pain when the wrist was stimulated.

### Beta activity during finger movement and electrical stimulation

No significant difference between patients and controls was found in the peak frequencies of movement-related beta ERD/ERS and electrical-induced beta ERS. The peak frequencies of the movement-related beta ERS were 20.1±0.5 Hz in controls and 19.3±1.0 Hz in patients. The peak frequencies of the movement-related beta ERD were 20.8±0.9 Hz in controls and 21.4±0.9 Hz in patients. The peak frequencies of the electrical-induced beta ERS were 18.8±0.7 Hz in controls and 19.2±1.2 Hz in patients.

No significant differences were found when comparing the peak latency of beta ERS either in finger lifting task (754±53 ms in controls; 932±93 ms in patients; *p*>0.05) or after electrical stimulation of median nerve (714±199 ms in controls; 712±127 in patients; *p*>0.05). There was no significant difference in the peak latency of movement-related beta ERD between controls and patients (10±190 ms in controls; 170±510 ms in patients; *p*>0.05).

**Figure 2:**
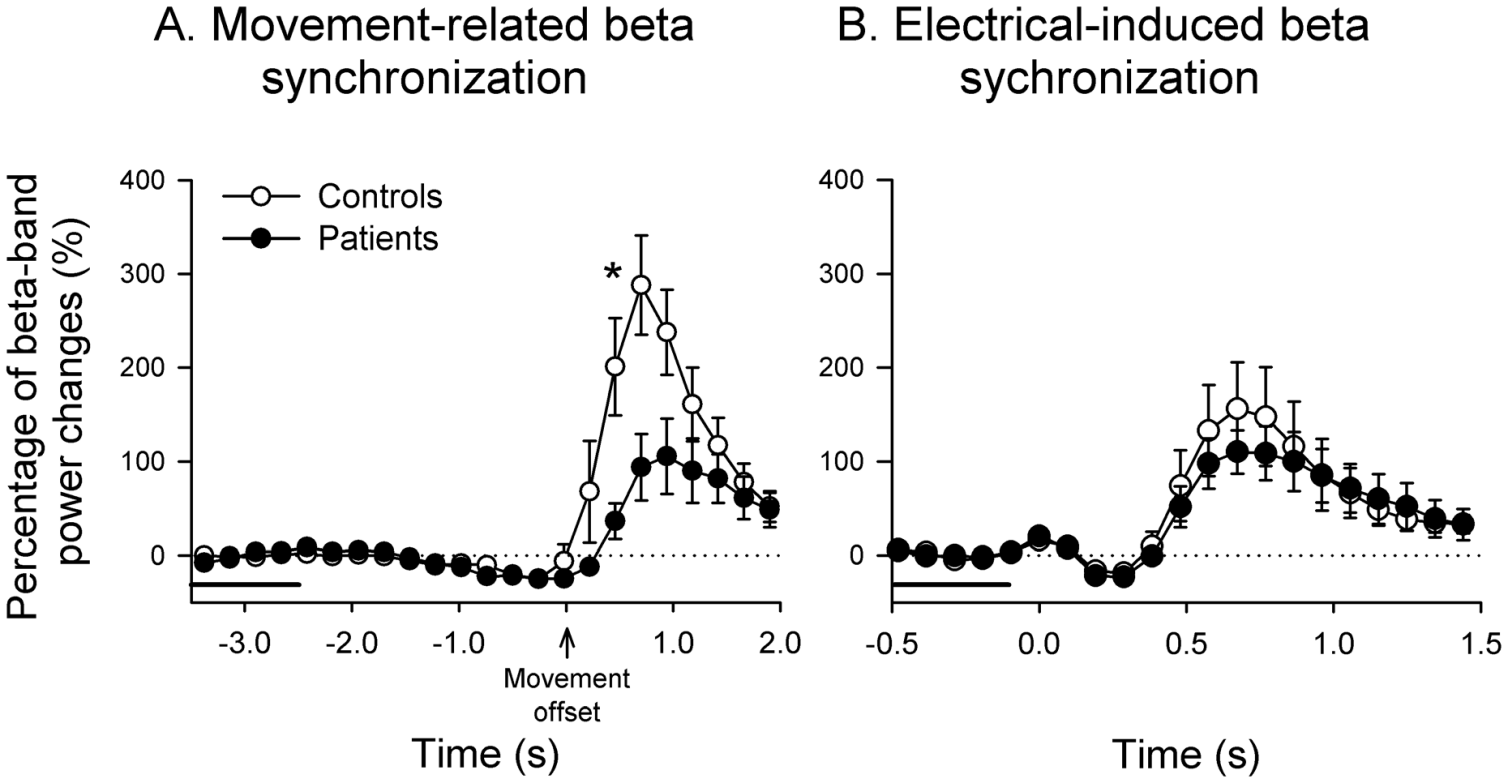
Movement-related and electrical-induced beta synchronization. **A**. The relative changes of movement-related beta power for controls (hollow circle) and patients with writer's cramp (solid circle) during the time interval from −3.5 to 2 s relative to movement offset. The horizontal line represents the reference period (3.5 to 2.5 s before the EMG offset). **B**. The relative changes of electrical-induced beta power for controls (hollow circle) and patients with writer's cramp (solid circle) during the time interval from −0.5 to 1.5 s relative to movement onset. The horizontal line represents the reference period (0.5 to 0.1 s before the electrical stimulation). Time zero indicates electrical trigger onset. Each scatter and error bar illustrates the mean (±SEM). The asterisk (*) denotes a *p* value of <0.05.

**Figure 3:**
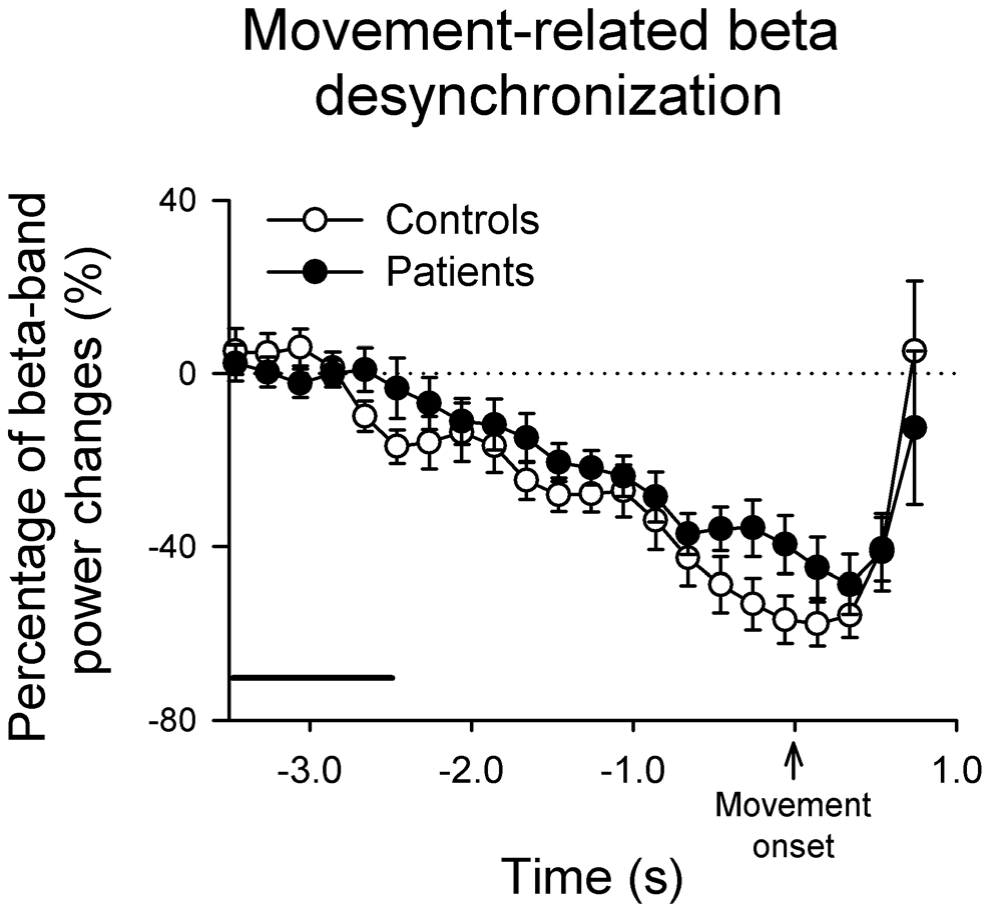
Movement-related beta desynchronization. The relative changes of movement-related beta power in controls (hollow circle) and patients with writer's cramp (solid circle) during the time interval from −3.5 to 2 s relative to movement onset. The horizontal line represents the reference period (3.5 to 2.5 s before the EMG onset). Each scatter and error bar illustrates the mean (±SEM).

The beta activities changed primarily over the semsorimotor area which is contralateral to the finger movement or electrical stimulation side. The beta ERS in patients was significantly diminished compared to control subjects around 0.5 seconds after the termination of finger movement (Fig. 2A). In contrast, the amplitude of electrical stimulation-induced beta ERS in patients with WC was comparable to control subjects (see Fig. 2B). For the amplitude of movement-related beta ERD, no differences were found between groups (Fig. 3).

## Discussion

In present study, we recruited patients with WC who manifest only when they are writing and not during other manual tasks. This study avoided the difficulty that occurs when interpreting data obtained by comparing WC patients with normal controls when there is different movement performance between the two groups. The analysis of beta oscillatory activity revealed a decreased inhibition of the contralateral motor cortex after movement termination in patients with WC as reflected by reduced beta ERS. Notably, the SEFs and sensory induced beta ERS were similar in the two subject groups. These findings suggest that there is a motor dependent dysregulation of sensorimotor cortical reactivity among patients who suffer from WC.

A main limitation in previous studies of movement-related alterations in dystonic patients has been the difficulties associated with interpreting data when there are different movement performances between the patients and control subjects. As writer's cramp is a task-specific dystonia, only writing or similar motor activities induces the symptoms. In the present study, the manifestations of WC in the patients included in this study are heterogeneous. All patients did not have any symptoms when carrying out the finger lifting task and undergoing median nerve stimulation. Therefore, the present study has the opportunity to observe any alterations that are caused purely by the disorder, namely writers' cramp.

Beta oscillation can be modulated by processing within the sensorimotor system. An increase in beta oscillation power has been found to occur under several different sensorimotor circumstances, including the termination of voluntary movement [8], [9], passive movement [13], imagined movement [24] and even tactile stimulation [10], [11]. The source of post-movement beta ERS has been found to be within the primary motor cortex [8]. The inhibitory neurons have been found to be important to the generation of beta-band synchronization. This generation of beta-band oscillations has been linked to the presence of inhibitory neurotransmitter [25]. Using transcranial magnetic stimulation, Chen and colleagues have demonstrated the time course of decreased corticospinal excitability after movement termination [26]. Therefore, it can be hypothesized that beta ERS reflects the deactivation/inhibition state of the motor cortex during the recovery phase of the movement process. Sensory induced beta ERS resembles those induced by voluntary movement but with a narrower range of peak frequencies [10], [11], [27]. The sources of sensory induced beta ERS are organized motorotopically in the primary motor cortex [28]. The primary motor cortex can be also activated by tendon vibration [29], suggesting a neural linkage between somatosensory feedback and motor output. The synchronized beta oscillation has been found to play a role in a large-scale sensorimotor network [30].

Previous neuroimaging studies have reported abnormalities in the motor cortex before movement execution in task-specific focal hand dystonia [31], [32]. In present study, this malfunction was also found after movement termination in patients with WC. However, beta oscillatory activities can be modulated by motor efflux from the motor system [24] or by the processes of the somatosensory afferent inputs [13]. Our data showed reduced movement-related beta ERS but a normal sensory induced beta ERS among patients with WC. In addition, the SEFs of patients were found to be comparable to those of the healthy controls. Thus the reduction in movement-related beta ERS in the patients might be related to an altered processing of motor activity rather than peripheral sensory afferents. Our data did not show a deficiency in the movement-related beta ERD before EMG onset in patients with WC. Beta ERD have been reported to be generated in the post-central gyrus [8], [11]. The electrical stimulation-induced beta ERD was also similar between patients and controls. Again, the results indicate the primary somatosensory cortex is intact in patients with WC.

Lack of inhibition has been found in patients with different types of dystonia [33]. The motor system uses a variety of forms of inhibition to control the precision and smoothness of movement. Such an inhibition is particularly important for a writing task as the intended finger movements requires contraction of specific muscles and selective inhibition of uninvolved ones. Although there is no overt structure neurodegeneration in WC, there may exist subtle changes in neuronal function and microstructural brain [34], [35]. A significant decrease in GABA level has been observed in the sensorimotor cortex of patients with WC at rest [34]. The evidence suggests that the resting level of beta oscillation is enhanced by an increase GABA-mediated inhibition in human sensorimotor cortex [25] and the GABA level in the motor cortex correlates with the amplitude of movement-related beta ERS [36], [37]. Therefore, we speculated that the reduction in movement-related beta ERS may be related to the deficient GABA level in patients with WC. This phenomenon is inherent in the patients with WC, even they did not perform a dystonia-inducing task. The loss of inhibition after termination of motor commands might contribute to the dystonic movements associated with WC.

Neruoimaging studies have shown normal electrophysiological processes in patients with dystonia [7], [32], [38]. In line with previous studies [7], [32], [38], the present study shows normal N20m and P35m responses in patients with WC. SEFs represent the responses of cortical neurons to changes in afferent activity [39]. This implies that the somatosensory processing from the peripheral to primary somatosensory cortex in patients with WC is relatively preserved. However, loss of somatosensory inhibition at spinal and cortical levels in patients with WC was discovered under paired-pulse with an interstimulus interval of 5 to 40 ms [40], [41]. In present study, the interstimulus interval is 3 s. The N20m and P35m deflections might have been able to recover fully after each stimulus after such a time interval [42]. Also the inter-subejct variation might be the other reason for the discrepancy [43]. In contrast with previous findings obtained within a various types of dystonic subject groups [40], we did not find abnormality in somatosensory responses in terms of SEFs and sensory induced beta ERS in our patients. These might be the reason why we did not detect any abnormality in the somatosensory evoked responses of the patients with WC.

The present study has some limitations. First, the sample size of the study is small. Studies with a larger sample size are needed to give a more comprehensive evaluation of changes in movement-related neural activities among patients with WC. Second, the simple finger task did not evoke dystonia symptoms. One may propose that the aberrant ERS data in WC patients are not so directly related to the appearance of hand dystonia. However, it seemed appropriate to use such a non-dystonia-inducing task in this study because the simple index-finger movement did not carry the confounding effect of excessive muscle activities and thus allowed for an objective comparison of cortical activations between patients and control subjects.

## Conclusion

A reduction in the movement-related beta ERS over the contralateral motor area in patients with WC is consistent with motor cortex dysfunction. However, no difference in sensory processing was found between WC patients and healthy controls. Our findings suggest a reduced inhibition within the motor cortex in patients with WC.
